# An AI ethics framework for a trustworthy autonomous drone system to support battlefield casualty triage

**DOI:** 10.1007/s43681-025-00967-3

**Published:** 2026-02-04

**Authors:** Peter Lee, Tasweer Ahmad, Syed Mohammad Waheed, Andrew Kenning

**Affiliations:** 1 Centre for Defence, Risk and Resilience, University of Portsmouth, Portsmouth, United Kingdom; 2 Department of Computer Science, Edge Hill University, Ormskirk, United Kingdom; 3 Advanced Engineering Centre, University of Brighton, Brighton, United Kingdom

**Keywords:** Ethical challenges, Robotic autonomous system, Post-trauma care, Ethical principles, AI-powered autonomous systems, Socio-technical property

## Abstract

AI-enabled capabilities in war provide new ethical challenges, even for nonlethal support tools such as the battlefield casualty triage drones that are the focus of this paper. We address an important and underexplored problem: how to embed ethical considerations into military AI systems that are designed to save lives rather than take them. The paper examines the ‘ATRACT’ project, which is developing an AI-powered drone as a trustworthy robotic autonomous system (RAS) to help frontline medics prioritise casualties in the critical post-trauma minutes that shape survival chances. As a position paper written while development is still underway, it includes the bespoke ethics framework created in the course of the project to date and offers real-time insights for other defence and security projects seeking to operationalise abstract AI ethics principles into concrete design and assurance guidance. We examine and draw upon approaches to operationalizing abstract principles in adjacent domains, to show how high-level principles can be translated into implementable requirements for technical robustness, ethical compliance, safety, and legal conformity, actively shaping system architecture, data, and human–machine interaction. We argue that trustworthiness is a socio-technical property that emerges from governance, documentation, and oversight rather than code alone, and that ethical assurance for triage drones must be designed in from inception and verified through ongoing testing, audit, and transparent evidence of due diligence.

**Supplementary Information:**

The online version contains supplementary material available at 10.1007/s43681-025-00967-3.

## An AI ethics framework for a trustworthy autonomous drone system to support battlefield casualty triage

Developing AI-driven autonomous systems for Defence poses profound ethical challenges. The aim of this position paper is to examine key aspects of an emerging ethics framework for an AI systems project in progress, to potentially be used as an exemplar for practitioners in related fields where ethical failures have severe consequences. The paper does this by examining the underexplored problem of how to embed ethical considerations into a military AI system that is designed to save rather than take lives. In this case, a battlefield casualty triage drone. While much academic and public debate around military AI focuses on autonomous weapons, support systems that use AI to save lives raise similarly complex issues of trust, accountability and compliance with humanitarian norms. A triage decision-support drone assisting a medic in the chaotic aftermath of a soldier being injured must support the operator in making data-based life-and-death decisions under battlefield pressures. If poorly designed or governed, such technology could erode human trust, introduce bias around whom it helps or prioritises, or inadvertently violate medical ethics or laws of war [19, 65]. Ethical foresight is therefore required. Emerging AI-enabled capabilities in war provide new ethical challenges that cannot be simply tagged on as an afterthought when equipment is deployed for use. The ATRACT project is still progressing, but the aim of this paper is to demonstrate—during the process—how and why ethical considerations [82] have been part of engineering design and development from the outset, to ensure the drone system is technically robust, operationally effective, ethically compliant and lawful.

A key stakeholder in this EPSRC-funded project is UK Defence Medicine, part of the UK Ministry of Defence (MOD). Consequently, one aspect of the approach taken in this project is to incorporate, as a minimum, UK MOD AI Ethics Principles to guide development and use of autonomous systems [48]. These five principles—Human-Centricity, Responsibility, Understanding, Bias and Harm Mitigation, and Reliability—emphasise that AI-based systems should prioritise human life and wellbeing, even in a military context, maintain accountability, explicability, enable transparency, and mitigate bias, while remaining robust and secure. Aligning the triage drone with the MOD’s principles ensures the system not only meets technical requirements but also reflects the values and legal standards that the military and society expect of a life-saving technology. In addition, medical ethics will be a key element of decision-making in this system, though it will remain the responsibility of the medic on the ground who receives casualty information via the drone, rather than being delegated to the drone itself [59].

High-level principles alone do not automatically translate into ethical processes or outcomes. A recurring theme in the AI ethics literature is the difficulty of moving from abstract principles to concrete practices [52, 78]. In response to these gaps, this project introduces the ATRACT Project Ethics Checklist as a concrete, operationalisable tool that bridges the gulf between high-level principles and day-to-day project development, and is set out at Appendix A. This checklist is a bespoke ethics framework, a work-in-progress integrated into the project from its inception [41]. It translates the MOD’s AI Ethics Principles—and a wider array of ethics principles—into implementable criteria for roboticists, AI developers and computer vision specialists to systematically apply, and update where necessary, throughout the drone’s development. It also draws from technically-oriented validation approaches from engineering, which will, in turn, be tested during the testing phase in due course [10, 11, 37]. Once the project is completed, the ethics checklist can be reviewed in light of the success or otherwise of the ATRACT system, and revised where necessary. However, the project team seeks to provide a realistic representation of the process as it is happening, instead of publishing a retrospective, idealised framework upon completion.

The remainder of this paper elaborates on the following challenges that have emerged in the course of the project so far. Section 1 provides an outline of the ATRACT medical triage drone, setting its development in a real-world context, describing how the system is designed to operate, and how the ATRACT ethics framework emerged in the project so far. Section 2 examines methodological challenges in creating an ethics framework for a trustworthy autonomous drone in two ways. First, introducing two forms of normative ethics and how they shape the inclusion of AI ethics principles, bioethics and medical ethics into the AI-enabled system. Second, it considers approaches from other technical fields on how abstract principles can be operationalised. Section 3 focuses on human centricity and autonomy, clarifying that the drone is a support capability that does not replace medics’ judgment. Then Sect. 4 addresses Mission Drift and Ethical Implications of Problem Framing when developing an AI-enabled triage drone. Finally, the paper concludes by summarising the challenges in embedding practical ethics in an AI-enabled triage drone designed to support medics, while recognising risks like bias, mission creep, and over reliance, while delivering a reliable, effective, trusted, and auditable capability.

## ATRACT (A trustworthy robotic autonomous system to support casualty triage) overview

ATRACT is a multi-university project supervised and funded by UK Research & Innovation (UKRI) and the Engineering and Physical Sciences Research Council (EPSRC), and entails the design, development, and implementation of an AI-powered, drone-based robotic autonomous system (RAS) to assist frontline medics in prioritising and minimising casualties on the battlefield, and essentially using AI to save lives as part of the UK’s national security and defence strategy.

The project aims to enhance the speed and efficiency of triage in the critical post-trauma minutes that shape battlefield survival chances. It was prompted by recent developments in drone use, including in modern warfare [79], which have disrupted conventional military helicopter evacuation methods [67], thus reducing casualty survival rates, increasing the likelihood of medic mortality, and complicating the decision-making process for the frontline medics.

ATRACT focuses on four main objectives, all of which represent innovations in the use of trustworthy AI and RAS technologies to advance research and development for national security and defence, whilst also yielding socio-economic benefits.

The first stage involves the development of a RAS using advanced sensors to detect and locate injured soldiers using thermal and visual imaging data, whilst simultaneously collecting vital sign data. The second and third stages comprise the use of advanced multimodal AI-sensing and state-of-the-art algorithms for real-time monitoring of casualties’ injury severity and their vital signs, whilst communicating this data to medics to assist them in casualty prioritisation and effective triage management. Finally, the fourth objective focuses on real-time monitoring to enhance resource management and thus minimise the risk of frontline medics being attacked, by reducing the time they are exposed to enemy threat.

**Fig. 1:**
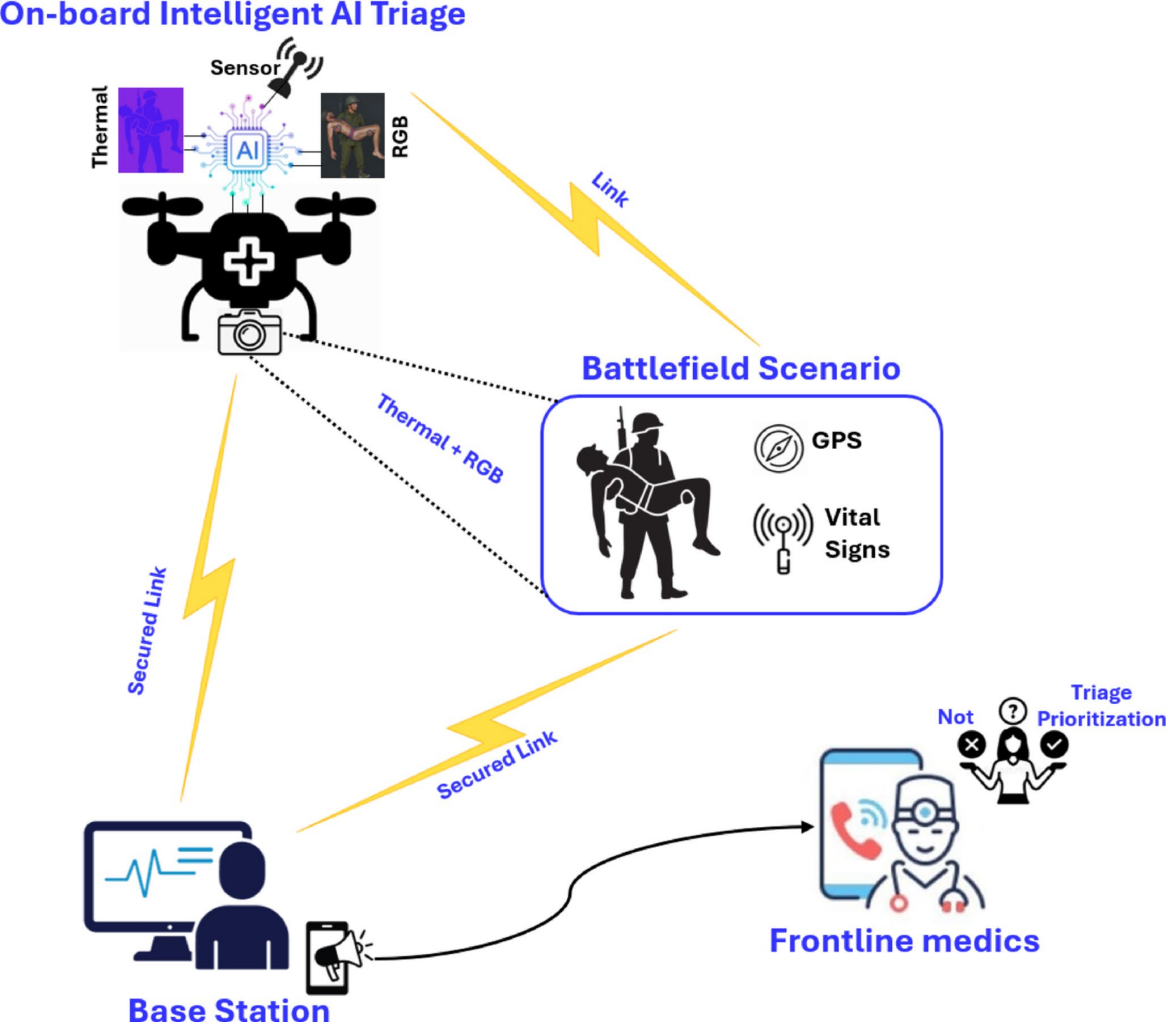
ATRACT AI-Powered Casualty Triage Workflow, where an AI-powered drone detects injured soldier in a battlefield scenario, carries out intelligent triaging and send signals to base station and medics for medical aid

Overarchingly, the triage process in the project entails the collection, transmission, and reception of primarily vital signs and GPS data from the soldiers (casualties) in the battlefield to the frontline medic on the ground, as well as medical teams at the command post. The project has trained advanced AI models and algorithms to process vital sign data post-telemetry. However, as seen in the final phase of the workflow in Fig. 1, the final decision with regard to prioritisation of the detected casualties and their subsequent treatment lies with the frontline medic(s). Accordingly, the configuration of AI tools, algorithms, and models collectively developed and deployed in this entire procedure is meant to augment the existing workflows and the overall job of a frontline medic, as opposed to replacing them altogether. In doing so, the “ethical principles for AI in defence” pertaining to *human-centricity* and *responsibility* [49] have been thoroughly addressed and catered to in the ATRACT project’s workflows.

## Bioethics, medical ethics and engineering solutions for decision support in an autonomous drone system for battlefield casualty triage

The ATRACT system comprises an AI-enabled drone element and a human medic who uses the triage drone information to support decision-making, and is assumed to make decisions in relation to two normative ethics frameworks: deontology and consequentialism. Deontology judges rightness by adherence to process, duties, rights, obligations and agent-centred constraints (such as not killing one innocent person to minimize overall killings) rather than by outcomes. Deontology emphasises intentions and means, rules and laws, and in some situations can forbid some outcome-improving actions [63, 74, 80]. In contrast, Consequentialism evaluates actions solely by the value of their consequences, or outcomes. This typically requires some form of maximising anticipated goods and is less concerned with the processes undertaken to achieve those goods [2, 42, 64].

**Fig. 2:**
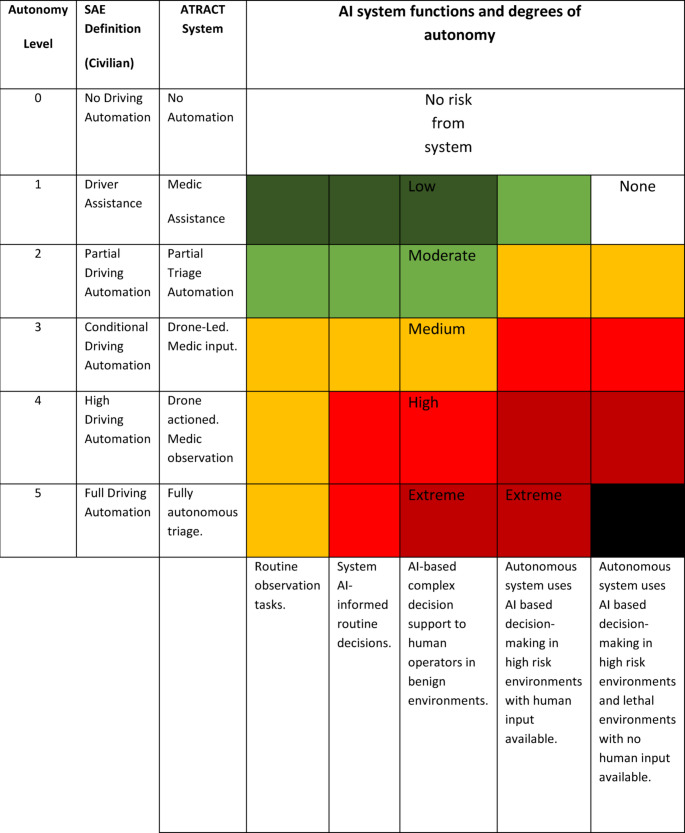
Risk matrix for AI-supported medical triage^1^

The ATRACT system combines predictable processes, powered by AI algorithms, with human operators—medics—who will exercise their judgement about if or how to incorporate the information provided by the drone element of the system. At that point, the medic is likely to make a consequentialist judgement in response to the following question: what will be the outcome for casualties identified by the drone, and where should care be prioritised? These judgments will carry varying degrees of risk, depending on the level of autonomy involved, the function of the system, and whether the operator becomes over-reliant on the AI’s recommendations [17]. Figure 2 sets out a risk matrix to represent the variables that impact risk at any point in the use of the system. It should also be noted that this ATRACT system is not intended to operate above Autonomy Level 3, and the AI-enabled drone element of the system will provide support to human decision making in a military-medical context, rather than take over the decision making [28]. It is anticipated, however, that future generations of similar systems will aim for higher levels of autonomy and for AI-based decisions in high-risk environments.

The ATRACT system ethics checklist incorporates established ethics principles and scholarship, such as the EU’s Assessment List for Trustworthy Artificial Intelligence (ALTAI) guidelines, the Institute of Electrical and Electronics Engineers’ (IEEE) Ethically Aligned Design [23, 38], and contributes to a rapidly developing field by providing a framework for AI practitioners that can be adapted and adopted as required. The checklist is implemented in a two-stage process: a short-form quantitative checklist to verify baseline compliance, followed by an in-depth qualitative questionnaire that prompts reflection and documentation of ethical reasoning [41]. This combination shifts the exercise from a tick-box compliance mindset within a process-driven framework, into an informed discussion embedded in the development workflow which also seeks to avoid negative outcomes and pursues positive outcomes.

The ethics checklist for an autonomous medical triage drone system must address both machine and human elements. The human elements concern the ethical decision-making of the practitioner or medic who uses the drone-sourced information. There are several layers of ethical consideration. This section expands on bioethical and medical ethics considerations required to be included in an ethics checklist for the development and production of a trustworthy autonomous drone used as a decision‑support tool by medics for battlefield casualty triage. The drone will operate under meaningful human control, never authorising or executing life‑or‑death clinical actions autonomously; responsibility for triage decisions remains with a suitably qualified medical person [26, 60, 72]. The intent is to translate widely‑used AI ethics and medical ethics principles into specific elements that shape design, deployment, testing and operations [23, 33, 52, 61]. The project team acknowledges, however, that future generations of triage drones are likely to have higher levels of autonomy and, potentially, full autonomy. The ethical challenges of those future fully autonomous triage drones are not addressed here.

### Operational ethics and responsibility

Ethical principles need to be translated into useable tools, checkpoints, and audit frameworks [6, 52, 61]. Embedded ethics, with ethicists and clinicians integrated into development and evaluation, help maintain value alignment in the context of their use [47]. From a requirement to be ‘ethical by design’ [13], with defence‑specific guidance bridging principles and doctrine [76, 78].

Responsibility is another key consideration. It will always be a human decision to deploy and use this medical triage drone in the context described here. Explicability is likewise shared between the human and the machine. On the technical side, there are challenges to demonstrate how the AI explains its recommendations and the processes by which it generates them. Separately, the user must be able to explain how and why they made particular decisions based on the drone’s recommendations. Finally, because the system is used for medical triage, both the human and machine components must conform to core biomedical principles.

The ATRACT checklist addresses four principles of biomedical ethics: beneficence, non‑maleficence, respect for autonomy, and justice. These are interpreted for war or other conflict settings [9, 45]. Beneficence and non‑maleficence entail demonstrable clinical benefit and harm‑minimisation; respect for autonomy motivates explainable outputs and clinician oversight; and justice requires fair performance across protected groups and operational contexts [53, 55].

In casualty triage, clinical need and the question of who should benefit guides prioritisation; any support tool such as the ATRACT drone, should reinforce this norm. Flexibility is required so that human clinicians can adapt recommendations to evolving tactical, resource, and clinical realities [46]. To reduce automation bias, the drone’s role is to broaden situational awareness without narrowing the judgement of the operator [26]. Anthropomorphism must be countered through interface design and training to prevent misplaced trust in ‘human-agent‑like’ systems. ‘Operator removal’—the progressive displacement of human judgement in the system by automation creep—should be acknowledged and risk‑managed in the training of operators [57].

### Trustworthiness and explicability

Trustworthiness is not a property of code alone but of sociotechnical practice, documentation, and oversight [43, 66, 77]. Trustworthiness is therefore dependent on the combined effects of accountability, closing responsibility gaps around design, deployment, failure‑response [73, 81], training and operational use. Given the high importance of clinical use, explicability—understood as a bundle of traceability, intelligibility, and justifiability—is essential [20]. It is difficult at this stage in the development of AI and autonomous systems to escape a double standard that tolerates a lack of clarity (or even failure) in human decisions while demanding full transparency and effectiveness of algorithms. Instead, a practical approach is to require task‑appropriate transparency for both [27]. Furthermore, the principle of Explicability can be used to demonstrate how a cross-disciplinary approach can also draw on established engineering practices.

### Engineering approaches

Technical validation methodologies have existed in the engineering field for many years. For example, the ATRACT system could have adapted usability engineering’s route from abstract principles to verifiable requirements by first defining the triage drone’s context of use: user roles (frontline medics), operational environment, time pressure, handover workflows, and failure consequences. With the context fixed in this way, ethical principles (explicability, human oversight, safety, bias mitigation, explainability) could be articulated as ‘Quality of use measures’ such as ‘effectiveness, efficiency and satisfaction with which specified users can achieve specified goals in specified environments’ [10, 11]. The Ethics Checklist is set out at Appendix A. Under the ‘Explicability’ heading it states:

*Explicability*

- A design-to-deployment auditing system is required for the AI elements.
- Ensure the following components of the ATRACT system are transparent;
  - Data sources, types of algorithms, learning techniques, AI models, and performance outputs.
  - Implement traceability for these elements and provide descriptions explaining what these are, the methods for developing them, and the reasons for these choices.
- Medical triage recommendations should be made in a transparent and accountable processes by;
  - Being explainable to the users.
  - Using traceable data sources, patterns, and algorithmic processes to review how and why recommendations were made.

There are both technical and human decision-making elements present, as there are for each of the ethics principles outlined in the Checklist.

However, a battlefield environment is a constantly changing physical context, and the ethical demands on the ATRACT system go beyond engineering quality. In one relatively benign context, the ‘quality’ of the system—which includes the medic who will make final triage decisions’—might be judged as ‘low’ if five out of ten casualties die. In contrast, in another context, saving only one out of ten casualties could be considered a success if they are under enemy gunfire. Therefore, while quality of the robotic part of the system—the drone—is important, it is a decision support tool rather than fully autonomous. The wider ATRACT ethical framework will remain as the basis for triage decision-making by the medic on the ground. Furthermore, since a key aspect of the project is to operationalise the MOD AI Ethics Principles, final decisions on use in the field will sit in the ethics space, rather than through technically-oriented validation alone.

Adopting a cross-disciplinary approach, insights from both the engineering domain and from normative ethics, are valuable. Looking ahead to the testing of the ATRACT system, the Common Industry Format offers a framework for validation and acceptance. Thresholds such as time-to-triage, decision-support accuracy for different types of wounds or injury, and operator workload, could be set out as auditable requirement statements tied to evaluation evidence [12]. ISO 9241-210’s lifecycle framing can anchor governance artefacts—context-of-use descriptions, risk logs, test reports, and change records—so each iteration shows what was tested, by whom, against which criteria, and what changed [37]. This approach yields traceability from principles to requirements to evidence, supporting independent assurance and continuous audit.

## Human-centricity & autonomy

This section focuses on human-centricity of AI implementation in battlefield triage scenarios, and the consequent degree of autonomy and agency that frontline medics possess over the decision-making process and outcomes, stemming from the information provided by an AI-powered drone. Subsequently, this section also offers an example to potentially be adopted or contextually improvised in similar AI-driven projects. Accordingly, this section begins by considering the ethical AI principle of human-centricity, then moves on to autonomy, showing how the ATRACT project can serve as an example for similar adherence to AI ethics in other projects.

i. Human-centricity:

The UK MOD’s Ethical Principles for AI in Defence states: ‘The impact of AI-enabled systems on humans must be assessed and considered, for a full range of effects both positive and negative across the entire system lifecycle’ [49]. To maintain human-centricity in this AI-enabled battlefield casualty-triage drone the project team has adopted a whole-lifecycle approach that considers the medics who will operate the drone, casualties, commanders, and affected civilians from design through development, testing, and evaluation. While this version of the drone will not be deployed, we have also considered issues around future deployment. At the design stage we have included stakeholder engagement, technical challenges and developments, and planned for human-AI interfaces, with traceability throughout [8, 24, 33]. Data governance ensures provenance, and in collecting images and videos with which to train the AI, we have sought to reduce the potential for bias by ensuring a wide range of, skin-tones, men and women, and different ages. Our data governance also supports auditability, in case we reach the testing phase and discover biased recommendations that undermine fairness [22, 24, 82].

Evaluation will seek to use mission-realistic scenarios (simulated casualty events), and user trials that verify human-in-the-loop arrangements. That is, the drone will provide clinical decision support by way of relevant information, while qualified personnel retain authority over triage categorisation, treatment, and evacuation prioritisation [5, 21, 48, 51]. The human–machine interface is intended to provide as much information as possible on the state of casualties, ‘civilian safety’ and an understanding of the context: danger levels; options for treatment; possibility of evacuation [21, 68, 83]. Documentation provides end-to-end traceability from design decisions and training data, to testing and evaluation behaviours and recommendations, enabling accountability and effective post-testing review [21, 33].

Preserving meaningful human control remains at the heart of the ATRACT project, even though the system is not a weapon platform. Incorrect decisions could still cost lives or increase human suffering. Continuous monitoring and feedback loops will be used to guard against model drift or shifting operational or ethical risks [21, 48, 51]. Throughout, compliance with international humanitarian law (IHL) protections for medical personnel will be assumed, with suitable emblems for conflict settings. Safety and risk management will be monitored throughout [9, 32, 83].

ii. Autonomy:

AI has many current and potential healthcare applications (drone-based) in battlefield settings, including triage, diagnosis, medical screening, resource allocation, and risk analysis, with differing complexity of task and levels of autonomy in each. Importantly, the varying degrees of autonomy that AI-powered drones possess include, but are not limited to: fully autonomous (human-out-of-the-loop), human-supervised (human-on-the-loop), or directly human-operated (human-in-the-loop) [18]. In a similar fashion, the implementation and form of AI applications in healthcare settings have been classified as “assistive AI”, where the final decision, albeit AI-augmented, is executed by the medic; and “autonomous AI”, where the AI makes the ultimate decision, minus the involvement of the medic [3, 44].

Empirical findings in existing research indicate that patients, owing to a range of factors including justice, trust, and overall satisfaction with the outcome [25], are more inclined to accept AI-automated decision-making for tasks that are more repetitive or “mechanical”, as opposed to decisions that require a more perceptive and empathetic insights, where human (medic) decision makers are preferred [40, 58, 69]. The aforementioned degrees of autonomy directly influence the extent of human agency and involvement [15, 16], and accordingly, must be cautiously and perceptively designed in order to avoid AI-powered development, implementation, and automation being conducted at the expense of ethical ramifications. A range of primary factors, including dehumanisation and decision-maker role appropriateness, have influenced differing perceptions of degrees of autonomy possessed by AI tools in healthcare applications [62, 70]. Accordingly, these factors along with the outcomes associated with varying levels of autonomy and agency, ought to be placed at the forefront of ethical frameworks governing the implementation of AI-powered tools in battlefield scenarios.

## Mission drift and ethical implications of problem framing

Problem framing is the foundational phase in the development of any AI system, especially critical in high-stakes applications, such as drone-based battlefield triage considered here. At this stage, defining the system’s intended purpose, stakeholders, operational scope, and success criteria establishes the ethical boundaries of its use. However, even seemingly life-saving, non-lethal technologies such as triage drones are susceptible to ethical pitfalls if the problem is framed too narrowly or without sufficient foresight. A primary concern is scope creep, where a system initially developed for medical assistance (e.g. battlefield casualties or noncombatants in the vicinity) could be adapted for surveillance, intelligence gathering, or even targeting—crossing the boundary from medical support to operational capabilities like intelligence gathering, surveillance, reconnaissance (ISR) or attack. This raises a dual-use dilemma, where the same sensors, algorithms, or decision-making logic could potentially be repurposed to support military operations [4, 75]. Such misuse of a medical-support technology would violate the Geneva Conventions—the Law of War—which states: that a medical unit or system loses protection and its use becomes unlawful if it goes beyond its humanitarian function and is used to commit ‘acts harmful to the enemy’ [30, 31, 34–36]. Such misuse would risk undermining the very trust and legitimacy that AI ethics principles aim to foster in a medical triage drone.

To mitigate these risks, the ethical principles Responsibility and Understanding, as outlined in the UK Ministry of Defence’s AI Ethics [48], have been used to shape system design. This includes clearly delineating acceptable uses, embedding safeguards to prevent mission drift, and building interpretability mechanisms so stakeholders understand what the system can and, equally importantly, cannot do. In practice, the ATRACT Ethical Checklist provides operational tools to translate these abstract principles into concrete practices by posing critical questions about dual-use implications and long-term accountability. AI systems, especially those used in conflict zones, must be designed with embedded foresight to avoid ethical erosion and mission repurposing that contravenes both IHL and wider societal expectations.

i. Ethics of data collection

In the ATRACT project, data collection for drone-based battlefield triage presents an ethically complex and technically delicate challenge. Battlefield environments are inherently chaotic, with constrained or no access to reliable, consent-driven data sources. Unlike commercial AI systems trained on open datasets, one option for the triage drone is to learn from imagery and vital signs captured in high-stakes, non-consensual, and often unpredictable scenarios. Obtaining informed consent from wounded combatants or civilians to use their images in a war context is impractical. Further, the individuals involved may be unconscious or in critical condition. This raises a clash of ethical priorities when individual human rights are commonly set aside in war, while autonomy and the right to privacy remain cornerstones of medical and AI ethics [8, 83]. A second option is the use of synthetic data, which brings its own challenges for training AI systems. Further, because of system safeguards, the project team could not use LLMs to generate images of wounds or injuries that could be used to train the ATRACT system.

Moreover, the risk of bias in the data—whether from uneven representation of ethnic groups, combat uniforms, or injury types—can directly influence who the AI system identifies and prioritises [7]. A drone trained disproportionately on images of light-skinned soldiers, for instance, may struggle to detect injuries on darker-skinned individuals, or vice versa, or misclassify culturally distinct attire as a threat. This reflects concerns by Floridi et al. [24] about ‘the risk of bias in datasets used to train AI systems’ where structural inequalities embedded in training data are silently amplified by technical systems. In the context of military triage, such biases could mean the difference between life and death, violating principles of justice and impartiality enshrined in both IHL and the Geneva Conventions.

To address these factors, the ATRACT project incorporates the Bias and Harm Mitigation and Justice principles from the UK MOD’s AI Ethics Framework [48] through critical scrutiny of data provenance, representation balance, and labelling practices. The ATRACT Ethical Checklist provides actionable prompts to evaluate the origin, diversity, and ethical integrity of collected data, ensuring that every image and signal contributes to a model that serves medics equitably and reflects the realities of the populations it is built to assist. In effect, data collection is not a neutral technical step but a normatively charged design decision—one that must be governed with the same rigour as model architecture or deployment logic. Failure to uphold these standards doesn’t just compromise performance; it endangers lives and undermines the legitimacy of AI systems in humanitarian applications.

ii. Balancing hardcoded and soft-coded ethical rules in AI systems

Hardcoded ethical rules refer to fixed, rule-based logic embedded into a system to enforce clear boundaries—such as “do not cross into human-occupied zones” or “halt operation if human detection confidence drops below a threshold” [1]. These rules provide safety where there are defined borders and clear targets—but often lack flexibility to adapt to nuanced or evolving situations. On the other hand, soft-coded or learned ethical behaviour—such as reward-based reinforcement learning or probabilistic decision-making—offers greater adaptability but can inadvertently generalise harmful patterns if training data is biased or incomplete [71].

The Amazon warehouse case illustrates this dilemma well [39]. Robots failed to detect workers wearing reflective vests due to training data limitations, and prioritised speed over safety in high-load conditions. A hardcoded rule to reduce speed when human presence is uncertain might have protected worker safety, but soft-coded performance tuning aimed at operational efficiency bypassed such safeguards. The system lacked a hybrid structure where immutable safety rules could coexist with adaptive productivity optimization.

A pragmatic approach lies in balance: hybrid systems where hardcoded “ethical hard stops” govern non-negotiable principles—like physical safety or Geneva Convention boundaries where they can be identified—while soft-coded modules handle contextual adaptation, such as dynamic path planning or crowd flow. Similar dilemmas exist in self-driving cars (e.g., Tesla FSD), where adaptive learning must be bounded by non-negotiable road safety laws. Developers must ensure that ethical boundaries are enforced not just through policy but through architecture—embedding logic-based constraints at system design level while allowing learning mechanisms to optimise within those constraints. The ATRACT system has the benefit of not employing a fully autonomous mode and still including a human medic in the system. However, future generations of triage drone are likely to move further towards full autonomy [50].

iii. Mission drift and ethical decay in real-world robotics

Ambiguity in defining acceptable use cases and the risk of model drift are closely linked ethical challenges, especially in real-world deployments like Boston Dynamics’ Spot robot. Initially designed for hazardous inspections in difficult-to-reach locations, Spot was later used for general surveillance and routine law enforcement patrols [56]. This represents *mission drift*—a form of ethical model drift—where the system’s actual use evolves beyond its originally intended and publicly accepted purpose. Because there were no hardcoded operational constraints (e.g., geofencing to hazardous zones, task-specific behavioural modes), the ethical boundary between humanitarian assistance and social surveillance blurred. In this context, ambiguity in use-case definition created the conditions for drift, and the lack of enforcement mechanisms allowed it to persist.

Similar cases reinforce the real-world prevalence of this issue. For instance, Clearview AI’s facial recognition software was initially marketed for law enforcement but was later found to be used by private companies and foreign governments—well beyond its original scope. Likewise, in healthcare AI, some triage systems trained on emergency room data began deprioritising patients from underrepresented demographics due to drift in real-time feedback loops [29]. These examples show that without clear boundaries encoded at both the policy and system architecture level, even well-intentioned AI systems can evolve toward ethically problematic applications. To counter this, organisations must implement both upfront use-case scoping and long-term drift monitoring—ensuring systems remain aligned with their intended mission and societal expectations.

iv. Bridging the gap: explainability and sim-to-real transfer in autonomous AI systems

Tesla’s Full Self-Driving (FSD) beta illustrates the explainability gap inherent in complex autonomous systems. While marketed as near-fully autonomous, FSD often makes seemingly inexplicable decisions, such as sudden braking or unexpected lane shifts, without providing drivers with clear reasoning. This opacity has led users to overtrust the system, believing it fully controls the vehicle, which has resulted in dangerous complacency and even fatal outcomes [14, 54]. From an ethics standpoint, AI-powered robotics must incorporate interpretable feedback mechanisms, like real-time alerts or visual/saliency cues from sensors, to help users understand why certain decisions are made. Without such transparency, users may misinterpret the system’s intent and misuse it, potentially leading to severe consequences.

Furthermore, the sim-to-real transition underscores another ethical technical challenge in Tesla’s deployment. Tesla extensively tests and refines its systems in simulated environments and limited real-world pilot zones, yet these controlled settings often fail to capture edge-case scenarios like phantom braking in low lighting or rare obstacle configurations. When FSD is released broadly, discrepancies emerge—such as unexpected braking or risky manoeuvres—that the system was neither trained nor stress-tested against. This exposes the need for rigorous sim-to-real validation pipelines, including scenario fuzzing, stress-testing models on ethically challenging edge variants (e.g., nighttime pedestrian detection, emergency vehicle encounters), and implementing on-vehicle fallback strategies that pause and alert humans when confidence is low. Without these checks, real-world deployment risks undermining both safety and public trust.

v. AI-driven impersonation and communication integrity in battlefield triage systems

AI-powered impersonation through deepfake voice cloning, synthetic text generation, and spoofed communication channels, presents a threat to the reliability and safety of drone-based battlefield triage. In such an environment, attackers could potentially mimic commanding officers, medics, or mission controllers, issuing falsified voice or text commands that redirect drones, alter casualty prioritisation, or override safety protocols. These fabricated communications may be indistinguishable from genuine orders, especially if transmitted in real time over secure-appearing channels.

The operational consequences are severe. False commands could mislead triage drones into bypassing critically injured individuals, entering hostile zones, or abandoning active rescue efforts, potentially violating IHL. In extreme cases, malicious actors might use impersonated alerts to create confusion among medics. Claiming, for example, that a zone is cleared when it remains dangerous, thereby undermining both mission effectiveness and trust in the system. Once such trust is eroded, operators may become hesitant to act on legitimate commands, delaying care during the “golden hour” after a wounding or injury when rapid intervention is most critical.

Mitigation requires a multi-layered approach. First, all voice and text inputs to the triage system should undergo cryptographic authentication, ensuring that only pre-verified devices and personnel can issue operational commands. Real-time deepfake detection tools must analyse voice spectral patterns and linguistic anomalies to flag suspicious inputs before execution. Second, hardcoded ethical boundaries such as operational geofencing and non-overridable casualty priority rules, should remain in force regardless of received instructions. Finally, comprehensive operator training and red-team exercises should familiarise personnel with impersonation tactics, reinforcing vigilance and adherence to verification protocols. Without these safeguards, AI-driven impersonation threatens not only the ethical integrity of battlefield triage systems but also the safety of those they are designed to protect.

## Conclusion

This paper has set out several of the ethical considerations that have been addressed in the design and development of an AI-enabled triage drone system to support medics in battlefield conditions, including practical approaches from engineering to ensure the quality and reliability of the drone. The project team’s aim is straightforward: to create trustworthiness through a combination of embedded AI principles, effective human-AI teaming, quality engineering, and sound decision-making by the human operator. We have considered human-centricity, responsibility, understanding, bias and harm mitigation, and reliability, all as part of an extensive ATRACT ethics checklist.

This paper has discussed several of these challenges as we continue to develop the system. We recognise that there are risks associated with bias, automation creep, mission creep, and the potential for either over-reliance or under-reliance by medics on this system. While the drone system has not yet reached the testing phase, work to address these elements remains ongoing.

The drone will remain a decision-support tool and will not be a decision-maker, serving to augment rather than replace human judgment. As the project matures, the ethical considerations of design, deployment, and use will continue to evolve through iterative reflection, evaluation, and testing. In summary, by making ethics actionable, we aim to deliver a system that is reliable, effective, trusted, and auditable. Future testing of the prototype will also include an examination of the ATRACT project ethics checklist, to assess the effectiveness of theoretical and practical assumptions during the development process. These will be reported in a subsequent paper.

## Supplementary Information

Below is the link to the electronic supplementary material.

Supplementary file1

## Data Availability

No datasets were generated or analysed during the current study.

## Footnotes

1 Cite as: Peter Lee, 2025, adapted from Peter Lee, ‘Risk Matrix: Autonomous Vehicles & Autonomous Weapon Systems’ in Ishmael Bhila, Peter Lee and Alison Wakefield. 2024. Autonomous Vehicles: Threats, Risks and Opportunities. ASIS Foundation Report 30
