# Supplementary material for: An AI ethics framework for a trustworthy autonomous drone system to support battlefield casualty triage
Source: AI Ethics. 2026 Feb 4;6(1):139. doi: 10.1007/s43681-025-00967-3 (PMC12868081; doi:10.1007/s43681-025-00967-3)
Supplement: Supplementary file 1 — Supplementary file1 [file 43681_2025_967_MOESM1_ESM.docx]

**APPENDIX A**

**ATRACT Project AI Ethics Checklist**

**(ATRACT: A Trustworthy Robotic Autonomous Drone System to Support Battlefield Casualty Triage)**

**Authors: Peter Lee; Andrew Kenning; Tasweer Ahmad; Syed Mohammad Waheed**

**Introduction**

The proliferation of Artificial Intelligence (AI) has produced calls for good governance and regulation, with governments and non-government organisations creating their own guidelines, frameworks and self-assessment toolkits for developing these technologies. Such attempts feature guidance for legal compliance and the construction of ethics principles influencing every stage from design-to-deployment [30]. This addition is important because, “AI [technologies] that are merely legally compliant will not necessarily be ethically justifiable or socially acceptable” [19]. Accordingly, an extensive range of AI ethics literature has emerged with a key study by the Berkman Klein Centre [5] identifying 36 ethics principles within 8 overarching categories which represent the most predominantly used concepts such as ‘accountability’ ‘explainability’ ‘responsibility’ and ‘non-discrimination’.

However, the main challenge for AI ethics is the operationalisation of ethics principles, because theoretical discussions regarding principles cannot address the complex issues produced by AI, and guidance is required for its lifecycle [26]. Therefore, there is a requirement for real-world applications which consider AI ethics from design-to-deployment as society moves from the *what* to the *how* of ethical implementation [26; 19]. In response, as of 2019, there were over 100 ethical frameworks created for AI [14; 21], and since then there has been further contribution to this field [2; 13]. Importantly for the political, social and cultural context of the ATRACT system, despite publishing guidance on AI, at the time of writing the UK has not developed a substantial, implementable framework to help govern ethical approaches or outline legal frameworks for developing AI technologies.

The UK Ministry of Defence (MOD) attempted to rectify this absence for Defence by developing *Ethical Principles for AI in Defence* and identifying *Key Challenges to Developing AI* [17]. Therefore, the ATRACT project presents a case study approach for translating these principles into an implementable framework that operates as a bespoke ethical checklist from the design-to-development of the drone system. The five UK MOD AI ethics principles are interpreted in relation to system requirements throughout the ATRACT system’s lifecycle which are centred on the UK MOD’s *key challenges* and how the project alleviates these concerns [19]. However, to ensure the *trustworthiness* element of ATRACT, the project aims to set itself apart and build upon these Defence-focused principles by incorporating two further principles: beneficence and justice. In addition, validation approaches from engineering guide the practical questions around drone development. Practically, the ATRACT checklist is completed through two stages to validate the ethics principles which underpin the system; a quantitative checklist that presents a short-form document to summarise whether key ethics matters have been addressed; and a qualitative checklist which requires significant detail to create an ethics audit for the AI developers [30].

**DISCLAIMER:**

**The ATRACT system is not fully autonomous: it is an AI-enabled, advanced decision-support system. Ultimate legal and ethical judgements are the responsibility of the human operator.**

This ATRACT Project ethics checklist is made available to inform public debate and provide insights for others who are developing AI-enabled technologies. This checklist should not be considered final. As the project develops, new insights and challenges will emerge and require ethical consideration, prompting amendments to this checklist. In a rapidly developing field the authors welcome comments and feedback. The project team requests that if you use or adapt this checklist for another project, that you acknowledge this work. Neither this checklist or any material in this document constitutes legal advice.

**Acknowledgement:** This project is funded by the UK Engineering and Physical Sciences Research Council Grant Ref: EP/X028631/1.

**ATRACT Project Artificial Intelligence Ethics Checklist**

**Summary**

| **Date** | **Work Package No:** | **Section Heading** | **Partial** | **Yes** | **No** | **N/A** |
| --- | --- | --- | --- | --- | --- | --- |
|  |  | 1. Human Centricity |  |  |  |  |
|  |  | 1. Responsibility |  |  |  |  |
|  |  | 1. Understanding |  |  |  |  |
|  |  | 4. Algorithmic Bias and Harm Mitigation |  |  |  |  |
|  |  | 5. Reliability |  |  |  |  |
|  |  | 6. Beneficence (benefitting users and society) |  |  |  |  |
|  |  | 7. Justice |  |  |  |  |
|  |  | 8. Non-Maleficence |  |  |  |  |

Refer to each of the sections in the full document below and complete as required. Once each section has been addressed, annotate this table to indicate if it has been completed, partially completed, not completed, or the section is not applicable to the work being carried out at this time.

**ATRACT Ethics Checklist**

1. **Human-Centricity**

The Human-Centricity principle is premised on how AI positively or negatively impacts humans through their relationships and interactions with these technologies. In this respect, “The choice to develop and deploy AI systems is an ethical one, which must be taken with human implications in mind” [17]. Human-centricity requires consideration from design-to-deployment, with continuous monitoring to identify any impacts on its users [17]. The ATRACT project has identified requirements concerning **human autonomy**; **human rights**; and **environmental sustainability** as necessary considerations for operationalising this principle.

**Human Autonomy**

- When the ATRACT system provides information that impacts humans, ensure the following components are implemented;
  - Protocols to ensure operators and casualties understand the involvement of AI in decision-making.
  - Design features that enable explainability for how and why recommendations were produced.
  - The ability to appeal AI-generated information by repeating the triage process and updating casualty status.
  - Mechanisms to ensure human operators have meaningful control and final oversight.
- Provide transparency regarding the ATRACT system and the levels of human-machine interaction required to operate the drone;
  - Document the different AI elements within the ATRACT system.
  - Explain the AI learning methods used within these.
  - Explain the software and hardware decisions producing the human-machine interface.
  - Ensure humans are integral to the Verification and Validation of performance outputs
- Establish defined levels of autonomy and determine if users can toggle between them.
  - Clarify how and to what extent medics on the ground operate as ‘experts-in-the-loop’.
  - Provide operational instructions to mitigate how the ATRACT system could impact human autonomy by altering their judgement leading to overreliance or under-reliance.
- The ATRACT drone is intended to be beneficial to its users, upskills medics, and augments casualty triage practices.
  - Conduct a risk assessment on potential positive and negative impacts on humans when developing and deploying the drone.
  - Confirm that potential risks or unintended consequences have been considered and mechanisms to address these have been implemented.

**Human Rights**

- Ensure the drone does not have a Lethal Autonomous Weapons System mode.
- Evaluate whether the ATRACT system impacts human rights throughout its lifecycle.
- Ensure the ATRACT-informed medic complies with the relevant UK and international human rights laws.

**Environmental Sustainability**

- Ensure environmental impacts are considered and minimised from design-to-deployment.
  - Evaluate the environmental benefits and negatives of the ATRACT system.
  - Document how environmental sustainability has been considered throughout ATRACT’s lifecycle;

1. **Responsibility**

The Responsibility principle is centred on how traditional understandings of command responsibility or individual responsibility are increasingly blurred by AI-enabled systems which create complex layers of decision-making with various levels of AI input [17]. This raises concerns about accountability, transparency, and human control which require attention from design-to-deployment. Delegating any aspect of ethical decision-making influenced by AI requires measures to ensure that humans have meaningful control, rights are protected, and the system can explain how and why decisions are made [11; 25]. Therefore, this ethics principle is operationalised by the requirements of **explicability**, **data protection,** and **human oversight** which establish clear lines of responsibility and accountability for the ATRACT system [17].

**Explicability**

- A design-to-deployment auditing system is required for the AI elements.
- Ensure the following components of the ATRACT system are transparent;
  - Data sources, types of algorithms, learning techniques, AI models, and performance outputs.
  - Implement traceability for these elements and provide descriptions explaining what these are, the methods for developing them, and the reasons for these choices.
- Medical triage recommendations should be made in a transparent and accountable processes by;
  - Being explainable to the users.
  - Using traceable data sources, patterns, and algorithmic processes to review how and why the information was presented.

**Data Protection**

- Compliance with relevant UK laws and regulations.
  - AQuA, GDPR, and UK Data Protection Act
  - Additional resources of Data Ethics Framework and ICO guidance and toolkit.
  - Establish who maintains responsibility for these processes and their documentation.
- Obtain consent, where relevant, and record when required throughout the lifecycle.

**Human Oversight**

- Ensure humans maintain overall responsibility and that any processes in the ATRACT system without human input are clearly presented.
  - The role of the operator using the AI system is defined to ensure human responsibility.
  - Established an override mechanism or the ability for medics to reject information from the ATRACT system.
- Map the agents of responsibility from design-to-deployment.
  - Developers of different components within the AI systems; those who tested the drone; authority figures within these structures; and users of the ATRACT system.
  - Determine who is responsible for errors and overseeing fixes from development to testing.
- Receive the appropriate ethical approval, where appropriate, at each stage of the ATRACT project.
  - Design and develop the ATRACT system to enable third-party auditing with any required documentation being easily accessible.
- Consider future procurement features within the design and development stage.
  - Transparent approach from design-to-deployment of AI elements.
  - Ethical and technical risk assessment that includes any identified potential future concerns.
  - Mechanisms for decommissioning the ATRACT drone system; data storage and AI models.

1. **Understanding**

Concerns for AI technologies emerge from their apparent unintelligibility, particularly a lack of transparency about how decisions are made and the impact upon accountability for those decisions [10; 12]. *Understanding* is operationalised by the design and development of AI systems to render algorithmic decisions and outcomes intelligible to users and external parties, which, in turn, helps create trustworthiness [17]. The checklist is premised on **traceability** and **explainability**. These are preconditions for assuring *understanding* because they produce mechanisms for accountability and transparency within the ATRACT system [27]. These range from technical design choices for the system itself by creating mechanisms to translate how algorithmic decision-making works and enable auditing, to social aspects which clarify benefits and limitations of the technology.

**Traceability**

- Define each stage of ATRACT’s lifecycle.
  - Identify the potential ethical risks within each phase and how to mitigate them.
- The following elements should be traceable to operationalise transparency and accountability;
  - Methods for incorporating AI elements in the design and production of the ATRACT system are clearly documented.
  - Data sources and data collection methods.
  - Data Labelling and Processing.
  - AI algorithms.
  - The AI models; their design principles, training methods, and how they were produced.
- Establish mechanisms for traceability in the algorithmic decision-making processes;
  - Be able to trace which data were used to make recommendations for categorisation and prioritisation in casualty triage.
  - Trace which AI model or rules produced the recommendation.
  - Monitor the drone’s actual performance outputs against those intended outputs from its Verification & Validation process.
  - Ability to record and evaluate the recommendations of the ATRACT system.

**Explainability**

- Ensure each AI element of the ATRACT system is explainable.
  - Explainability to be assessed during testing to determine how interpretable AI decisions are to users.
  - The performance outputs are presented in user-friendly formats.
- Ensure the logic and rationale of the algorithmic decision-making are transparent.
  - Determine what data and AI models were used during casualty triage.
  - Users and external parties to be able to monitor how and why recommendations were produced.
- Create manuals or guidance for operating the ATRACT drone.
  - Ensure medics receive training on understanding the ATRACT system; the benefits and limitations of the drone; inputs required to assure and sustain its performance; and how to evaluate its outputs.
  - Document the expert feedback of medics and how their insight has been utilised.

1. **Algorithmic Bias and Harm Mitigation**

The ATRACT system must fairly recognise the diverse communities the drone operates within and not cause harm on the basis of race, sex or age as a result of its different AI elements affecting humans. This can be actioned by understanding how bias emerges in AI systems, establishing safeguards to mitigate harm, and implementing monitoring for accountability [16; 24]. The UK MOD requires developers to be, “Carefully curating and managing datasets, setting safeguards and performance thresholds throughout the system lifecycle, managing environmental effects, and applying strict development criteria for new systems, or existing systems being applied to a new context” [17]. The ATRACT project operationalises these practices through requirements of non-discrimination.

**Non-Discrimination**

- Define an understanding of fairness and non-discrimination and explain its application to the expected outputs of the ATRACT system.
- Ensure the ATRACT system performs fairly and does not discriminate on the basis of race, sex or age during casualty triage.
  - Data collection is appropriate for operational requirements.
- Develop mechanisms to identify potential biases throughout the lifecycle.
- Implement techniques to mitigate biases throughout the lifecycle.
- Ensure non-discrimination and fairness within the facial expression component of the ATRACT system.
  - Transparent and accountable approach from design-to-deployment for how this technique operates.

**Accessibility**

- Develop a user-friendly interface.
  - Outputs are easily accessible and explainable.
  - Implemented usability testing and document feedback.
  - Ensure accessibility is incorporated from design-to-deployment.

1. **Reliability**

The principle of reliability seeks to assure the consistent performance of the ATRACT drone system in different environments and contexts throughout its lifecycle, while creating a robust and secure approach to data protection that can mitigate damage caused by cyberattacks [2; 27; 17]. Operationalising reliability develops confidence that AI technologies, “Fulfil their intended design and deployment criteria and perform as expected, within acceptable performance parameters” [17]. *Reliability* plays an important role in developing trust with AI by forming a close relationship with *understanding*. Establishing monitoring systems helps demonstrate to users that the AI is constantly reviewed and there is predictability in its outcomes, which are traceable to determine if the technology is performing as required [11; 17]. Accordingly, the two requirements of reliability presented here are predictability and accuracy which help ensure the performance of the ATRACT system.

**Predictability**

- Evaluate if the performance of the ATRACT system is achieving intended outcomes.
  - Define expected and V&V outcomes.
- Implement mechanisms to mitigate failures in the performance of the drone.
  - Use field testing to confirm what ‘success’, ‘failure’ and ‘errors’ means in the system’s performance.
  - Establish ways to test and increase the reproducibility of the expected outputs.
- Ensure the accuracy and quality of any new data obtained during use of the system.
- Establish safeguards and security to prevent harmful effects through the following;
  - Design or technical faults from software or AI modelling; damage to and outages of the drone; and cyber-attacks.

**Accuracy**

- Ensure high-quality data is used.
  - Provide an explanation for the data sources and statistical methodologies used in collection.
  - Data sample is of appropriate quality and size to assure high performance.
  - Label data on the basis of non-discrimination.
- Monitor the accuracy of the drone’s outputs.
  - Record ‘positive,’ ‘false positives’, ‘false negatives’, etc.
  - Ensure these are human verified and validated.
- Evaluate the testing tasks so that any concerns can be identified and resolved.

1. **Beneficence**

Beneficence requires this AI-enabled system to be beneficial to its human users and to wider society [1]. Ethically, AI assistance or autonomous capabilities in medicine require the technology to be beneficial for all parties and not simply a cost-effective means of providing care [29]. In the context of the ATRACT system, beneficence means ensuring the well-being of the medics and casualties. Practically this entails making judgements about the benefits of deploying the drone [28].

- Transparent documentation of the ATRACT system; collection of training data; data labelling; AI modelling; algorithms.
- The expected and actual outputs of the ATRACT system impact positively on medics and casualties.
- Outline the expected positive outcomes and any negative impacts identified during development.
  - Highlight any technical or ethical issues; clarify if they have been resolved or need to be addressed at a later stage.
- ATRACT’s predictions to assist triage decisions must inform Defence medical ethics.
- The guidelines: ‘Ethical decision-making for doctors in the armed forces: a tool kit’ is to be used by medics to guide their triage practices when using the ATRACT system.
  - Additional checklist criteria to consider before deployment: The WHO Surgical Checklist; is the drone necessary?; is the operational environment suitable (for example, could use of the ATRACT drone system increase risk of enemy action against the users?); can the drone be used to advise on the operational environment and provide situational awareness to deal with casualties, without becoming an intelligence source and breaching Geneva Convention requirements?
  - Consider if or how consent factors into the ATRACT system and military triage protocols.
- Ensure expert feedback is obtained from medics throughout ATRACT’s testing.
  - Testing phases to provide first-hand expert advice on operating the drone.
- Conduct a safety hazard assessment for operating the drone or being in close proximity.

1. **Justice**

This principle relates to non-discrimination in providing medical care and impartial decision-making, where military triage prioritises and categorises casualties on clinical need and survivability [3; 7; 23]. Any harmful biases emerging from unrepresentative training data, algorithms, or learning techniques during deployment can exacerbate existing discrimination and unfairness for those requiring medical assistance [4]. This can potentially impact the medic’s decision-making and patient well-being. In that situation, deploying the drone could even be unethical and bring legal ramifications [22]. Furthermore, at this stage, AI is not being used to replace human judgment in medical triage ethics, even in war, which require the most seriously ill or wounded casualty to be prioritised. This could potentially be an enemy combatant or a civilian, known as ‘dual obligations’ within medical ethics and the Geneva Convention [7]. Therefore, the ATRACT system must take account of such complexities, while acknowledging the practical difficulties involved.

- The ATRACT system must operate fairly and not harm casualties through discriminatory practices.
- The ATRACT system should not infringe on human rights or disregard relevant legal frameworks and regulations. For example, a badly wounded civilian should be prioritised over a less seriously wounded allied combatant.
- Medics will remain responsible for complying with existing guidance on military casualty triage protocols.
- Medics will remain responsible for avoiding harmful bias affecting medical decisions.
- Data labelling should be conducted with fairness and non-discrimination in mind.
- Validation & Verification of ATRACT predictions must be performed by humans.
- Implement the following measures to operationalise human control and decision-making;
  - Ensure users can trace the decision-making process of the AI system.
  - Ensure mechanisms to reproduce triage assessment.
  - Ensure medics can override the ATRACT system if necessary.

Review performance and any potential unethical practices after testing.

1. **Non-Maleficence**

Non-Maleficence requires that the system does not do anything that can be considered evil in itself, including inflicting harm or injury on patients [3]. In the context of AI, non-maleficence refers to both how developers design the system and the technology itself from how it interacts with humans. It cannot be assumed that the ATRACT system will always ensure the well-being of medics and casualties throughout its lifecycle [8; 9]. From design-to-deployment the AI developers must consider how their design choices, approaches to data security, and monitor any features of the system that could potentially cause harm if safeguards are not in place [1; 27]. Therefore, the project must consider how the ATRACT system may cause harm from human-machine interactions. This includes potential bias, data privacy, and human dignity for casualties being processed through cameras, sensors, and algorithmic calculations [10; 15].

- Monitoring of the ATRACT system for harm must be performed by humans.
- Users of the ATRACT system must respect human dignity and understand the potential for dehumanisation.
- Establish safeguards and protocols for identifying and mitigating harmful unintended consequences when they occur.
  - Safeguards for the following; Providing location of these actors to the enemy; Electronic interference with drone or data; Environmental factors; Cases if/when the drone malfunctions.
  - Monitor and record these throughout the lifecycle.

**References**

[1] Adams, C., Penter, P., Lemermeyer, G., & Rockwell, G. (2023) ‘Ethical principles for artificial intelligence in K-12 education’. Computers and Education: Artificial Intelligence. Volume 4, 100131,<https://doi.org/10.1016/j.caeai.2023.100131>.

[2] European Commission (2020) ‘Assessment List for Trustworthy Artificial Intelligence (ALTAI) for self-assessment’. 17 July. <https://digital-strategy.ec.europa.eu/en/library/assessment-list-trustworthy-artificial-intelligence-altai-self-assessment> [Accessed 8 August 2025]

[3] Beauchamp, T.L. and Childress, J.F. (2013) Principles of biomedical ethics. 7th edn. Oxford: Oxford University Press.

[4] Benzinger L, Ursin F, Balke WT, Kacprowski T, Salloch S. Should Artificial Intelligence be used to support clinical ethical decision-making? A systematic review of reasons. BMC Med Ethics. 2023 Jul 6;24(1):48. doi: 10.1186/s12910-023-00929-6. PMID: 37415172; PMCID: PMC10327319.

[5] Berkman Klein Centre (2020) ‘Principled Artificial Intelligence: Mapping Consensus in Ethical and Rights-based Approaches to Principles for AI. January. <https://cyber.harvard.edu/publication/2020/principled-ai> [Accessed 12 May 2024]

[6] Blanchard, A., Thomas, C. & Taddeo, M. Ethical governance of artificial intelligence for defence: normative tradeoffs for principle to practice guidance. AI & Soc 40, 185–198 (2025). <https://doi.org/10.1007/s00146-024-01866-7>

[7] BMA (2012) ‘Ethical decision-making for doctors in the armed forces: a tool kit’. <https://www.bma.org.uk/media/zezp5luv/armed-forces-toolkit-updated-2025.pdf> [Accessed 23 June 2024].

[8] Cawthorne, D. and Wynsberghe, A. van (2020) ‘Artificial intelligence and military decision-making’, AI and Society, 35(4), pp. 905–916.

[9] Floridi, L. and Cowls, J. (2019) A unified framework of five principles for AI in society. Harvard Data Science Review, 1(1). <https://doi.org/10.1162/99608f92.8cd550d1>

[10] Floridi, L., Cowls, J., Beltrametti, M., Chatila, R., Chazerand, P., Dignum, V., Luetge, C., Madelin, R., Pagallo, U., Rossi, F., Schafer, B., Valcke, P. and Vayena, E. (2018) ‘AI4People—An ethical framework for a good AI society: Opportunities, risks, principles, and recommendations’, Minds and Machines, 28(4), pp. 689–707.

[11] French, S.E. and Lindsay, L.N. (2022) ‘Artificial Intelligence in Military Decision-Making: Avoiding Ethical and Strategic Perils with an Option-Generator Model’, in Emerging Military Technologies pp. 53–74, DOI: <https://doi.org/10.1163/9789004507951_007>

[12] Günther, Mario & Kasirzadeh, Atoosa. (2022). Algorithmic and human decision making: for a double standard of transparency. AI & SOCIETY. 37. 10.1007/s00146-021-01200-5.

[13] Huang, C., Zhang, Z., Mao, B. and Yao, X. (2023) ‘An Overview of Artificial Intelligence Ethics’. in IEEE Transactions on Artificial Intelligence. Vol. 4 (4) pp. 799-819. Doi: 10.1109/TAI.2022.3194503.

[14] Jobin, A., Ienca, M. and Vayena, E., 2019. The global landscape of AI ethics guidelines. Nature Machine Intelligence, 1(9), pp.389–399.

[15] Jotterand, F. and Bosco, C. (2020). ‘Keeping the “Human in the Loop” in the Age of Artificial Intelligence’. Science and Engineering Ethics 26 (5):2455-2460.

[16] Leslie, D. (2019). Understanding artificial intelligence ethics and safety: A guide for the responsible design and implementation of AI systems in the public sector. The Alan Turing Institute. <https://doi.org/10.5281/zenodo.3240529>. [Accessed 12 June 2024].

[17] Ministry of Defence (2022) Ambitious Safe and Responsible: Ethical principles for artificial intelligence in defence. London: UK Ministry of Defence. <https://www.gov.uk/government/publications/ambitious-safe-responsible-our-approach-to-the-delivery-of-ai-enabled-capability-in-defence> [Accessed 13 September 2023]

[18] Ministry of Defence (2022) Defence Artificial Intelligence Strategy. London: Ministry of Defence. Available at: <https://www.gov.uk/government/publications/defence-artificial-intelligence-strategy> [Accessed 13 September 2023].

[19] Morley, J., Floridi, L., Kinsey, L. and Elhalal, A. (2021) From what to how: an initial review of publicly available AI ethics tools, methods and research to translate principles into practices. Science and Engineering Ethics (2020) 26: 2141–2168.

[20] Morley, J., Machado, C., Burr, C., Cowls, J., Taddeo, M., & Floridi, L. (2019) The Debate on the Ethics of AI in Health Care: a Reconstruction and Critical Review. SSRN Electronic Journal. 10.2139/ssrn.3486518.

[21] Morley, J., Floridi, L., Kinsey, L. & Elhalal, A. (2020) From What to How: An Initial Review of Publicly Available AI Ethics Tools, Methods and Research to Translate Principles into Practices. Science and engineering ethics. 26. 10.1007/s11948-019-00165-5.

[22] Müller, Vincent C. (2021) Ethics of Artificial Intelligence. In Anthony Elliott, The Routledge Social Science Handbook of Ai. Routledge. pp. 122-137.

[23] NATO (2021) ‘Summary of the NATO Artificial Intelligence Strategy’. 22 October. <https://www.nato.int/en/about-us/official-texts-and-resources/official-texts/2021/10/22/summary-of-the-nato-artificial-intelligence-strategy> [Accessed 12 June 2024]

[24] Ntoutsi E, Fafalios P, Gadiraju U, Iosifidis V, Nejdl W, Vidal ME et al. Bias in data-driven artificial intelligence systems—An introductory survey. Wiley Interdisciplinary Reviews: Data Mining and Knowledge Discovery. 2020 May 1;10 (3):e1356. doi: 10.1002/widm.1356

[25] Schwarz, E. (2018). Technology and moral vacuums in just war theorising. Journal of International Political Theory, 14(3), 280-298. <https://doi.org/10.1177/1755088217750689> [Accessed 17 February 2024]

[26] Taddeo, M., Blanchard, A. and Thomas, V., 2023. Operationalising AI Ethics in Defence. Journal of Military Ethics, 22(1), pp.15–28.

[27] UNESCO (2022) Recommendation on the Ethics of Artificial Intelligence. Adopted on 23 November 2021. <https://unesdoc.unesco.org/ark:/48223/pf0000381137> [Accessed 14 April 2024].

[28] World Health Organization (2021) Ethics and governance of artificial intelligence for health. Geneva: WHO.

[29] Zardiashvili, L., Fosch-Villaronga, E. “Oh, Dignity too?” Said the Robot: Human Dignity as the Basis for the Governance of Robotics. Minds & Machines 30, 121–143 (2020). <https://doi.org/10.1007/s11023-019-09514-6> [Accessed 12 December 2024].

[30] Zhou, J., Chen, F. ‘AI ethics: from principles to practice’. AI & Society. 38, 2693–2703 (2023). <https://doi.org/10.1007/s00146-022-01602-z> [Accessed 12 December 2024].
